# Characterisation of small molecule ligands 4CMTB and 2CTAP as modulators of human FFA2 receptor signalling

**DOI:** 10.1038/s41598-018-36242-1

**Published:** 2018-12-13

**Authors:** Zoe V. Schofield, Daniel Croker, Avril A. B. Robertson, Nicholas L. Massey, Chantal Donovan, Ernest Tee, David Edwards, Trent M. Woodruff, Reena Halai, Philip M. Hansbro, Matthew A. Cooper

**Affiliations:** 1 Institute for Molecular Bioscience, The University of Queensland, Brisbane, Australia; 2 School of Biomedical Sciences, The University of Queensland, Brisbane, Australia; 3 The School of Chemistry and Molecular Biosciences, The University of Queensland, Brisbane, Australia; 4 Priority Centre for Healthy Lungs, University of Newcastle & Hunter Medical Research Institute, Newcastle, NSW Australia

## Abstract

Short chain fatty acids (SCFAs) are protective against inflammatory diseases. Free fatty acid receptor 2 (FFA2), is a target of SCFAs however, their selectivity for FFA2 over other FFA receptors is limited. This study aimed to functionally characterise 2-(4-chlorophenyl)-3-methyl-N-(thiazole-2-yl)butanamide (4CMTB) and 4-((4-(2-chlorophenyl)thiazole-2-yl)amino)-4oxo-3-phenylbutanoic acid (2CTAP), and their enantiomers, in modulating FFA2 activity. The racemic mixture (*R/S*) and its constituents (*R-)* and (*S-*) 4CMTB or 2CTAP were used to stimulate human (h)FFA2 in the absence or presence of acetate. Calcium ions (Ca^2+^), phosphorylated extracellular signal-regulated kinase 1 and 2 (pERK1/2) and cyclic adenosine monophosphate (cAMP) were measured. *R/S*-4CMTB is a functionally selective ago-allosteric ligand that enhances Ca^2+^ response to acetate. Both *R/S*-4CMTB and *S*-4CMTB are more potent activators of pERK1/2 and inhibitors of forskolin-induced cAMP than acetate. *S*-4CMTB increased neutrophil infiltration in intestinal ischemia reperfusion injury (IRI). 2CTAP inhibited constitutive Ca^2+^ levels, antagonised acetate-induced pERK1/2 and prevented damage following IRI. This study characterises enantiomers of functionally selective ligands for FFA2 in cells stably expressing hFFA2. It highlights the novel roles of selective FFA2 enantiomers 4CMTB and 2CTAP on Ca^2+^, pERK1/2 and cAMP and their roles as allosteric modulators which, may assist in efforts to design novel therapeutic agents for FFA2-driven inflammatory diseases.

## Introduction

Short chain fatty acids (SCFAs) impart protective effects in inflammatory diseases such as colitis, asthma and arthritis^1^. They act on many cell types, including neutrophils, principally by acting on free fatty acid receptors (FFARs). Free fatty acid receptor 2 (FFA2/FFAR2) is a classical G protein-coupled receptor (GPCR) that signals through Gα proteins. It is highly expressed on cells expressing intestinal peptide YY (PYY) and glucagon-like peptide-1 (GLP-1); *viz*. endocrine L-cells, basal crypts, colonocytes and enterocytes of the small and large intestine^2,3^. Most studies have probed the role of FFA2 in metabolic disorders^4,5^. However, the highest expression of FFA2 is found in innate immune cells, especially in neutrophils, eosinophils, monocytes and B-lymphocytes^2,6,7^. This suggests that the receptor may also have a role in innate immunity and inflammation. This has led to studies of FFA2 in inflammatory disorders such as inflammatory bowel disease^8^, colon cancer^9^, asthma^10^ and arthritis^11^. The role of FFA2 as a regulator of inflammation is further supported by observations of FFA2-deficient mice (FFA2^−/−^), which exhibit exacerbated or non-resolving inflammation in colitis, arthritis and asthma models^11^.

Acetate, propionate and butyrate elicit FFA2-dependent chemotaxis of immune cells, most notably neutrophils^12–14^. Conversely, FFA2^−/−^ mice are more prone to inflammation in DSS-induced colitis^12^. These potential discrepancies are likely due to the role of neutrophils in acute versus chronic situations. FFA2 shares 43% similarity with FFA3^15^ and both respond to the same SCFA ligands due to their highly conserved orthosteric sites. The order of potency for FFA2 is acetate (C2) = propionate (C3) > butyrate (C4) > valerate (C5) = formate (C1), and for FFA3 is butyrate (C4) = propionate (C3) = valerate (C5) > acetate (C2) = formate (C1)^6^. FFA2 and FFA3 have different tissue expression profiles and physiological roles, but there remains insufficient discrimination of SCFA binding to produce adequate drug candidates^7^. Additionally, SCFAs have poor potency and high μM-mM concentrations are required to elicit FFA2 and FFA3 responses *in vitro*, which has led to the search for more potent and selective synthetic ligands.

This study fully characterises two FFA2 ligands; *R-*, *S-*, *R/S-* 2-(4-chlorophenyl)-3-methyl-N-(thiazole-2-yl)butanimide (4CMTB) and 4-((4-(2-chlorophenyl)thiazole-2-yl)amino)-4oxo-3-phenylbutanoic acid (2CTAP), and probes their signalling profiles in CHO cells stably expressing the human FFA2 receptor (hFFA2). We demonstrate that racemic 4CMTB is an ago-allosteric ligand, enhancing calcium ion (Ca^2+^) responses to acetate. *R/S*-4CMTB and *S*-4CMTB are more potent activators of phosphorylated extracellular signal-regulated kinase 1 and 2 (pERK1/2) and inhibitors of forskolin-induced cyclic adenosine monophosphate (cAMP), compared to acetate alone. 2CTAP is an inverse agonist that inhibits constitutive Ca^2+^ levels and antagonises acetate-induced pERK1/2, but has no effect on cAMP. Conversely, at low concentrations both *R*- and *S*-4CMTB and *R*- and *S*-2CTAP are negative allosteric modulators of Ca^2+^-induced acetate responses. We report novel roles for the selective FFA2 enantiomers, 4CMTB and 2CTAP, on Ca^2+^, pERK1/2 and cAMP responses and as allosteric modulators and, highlight their activity in an *in vivo* intestinal ischemia reperfusion injury (IRI) model.

## Results

### Characterisation of *R/S*-4CMTB and *R/S*-2CTAP in CHO cells stably transfected with FFA2

**Figure 1:**
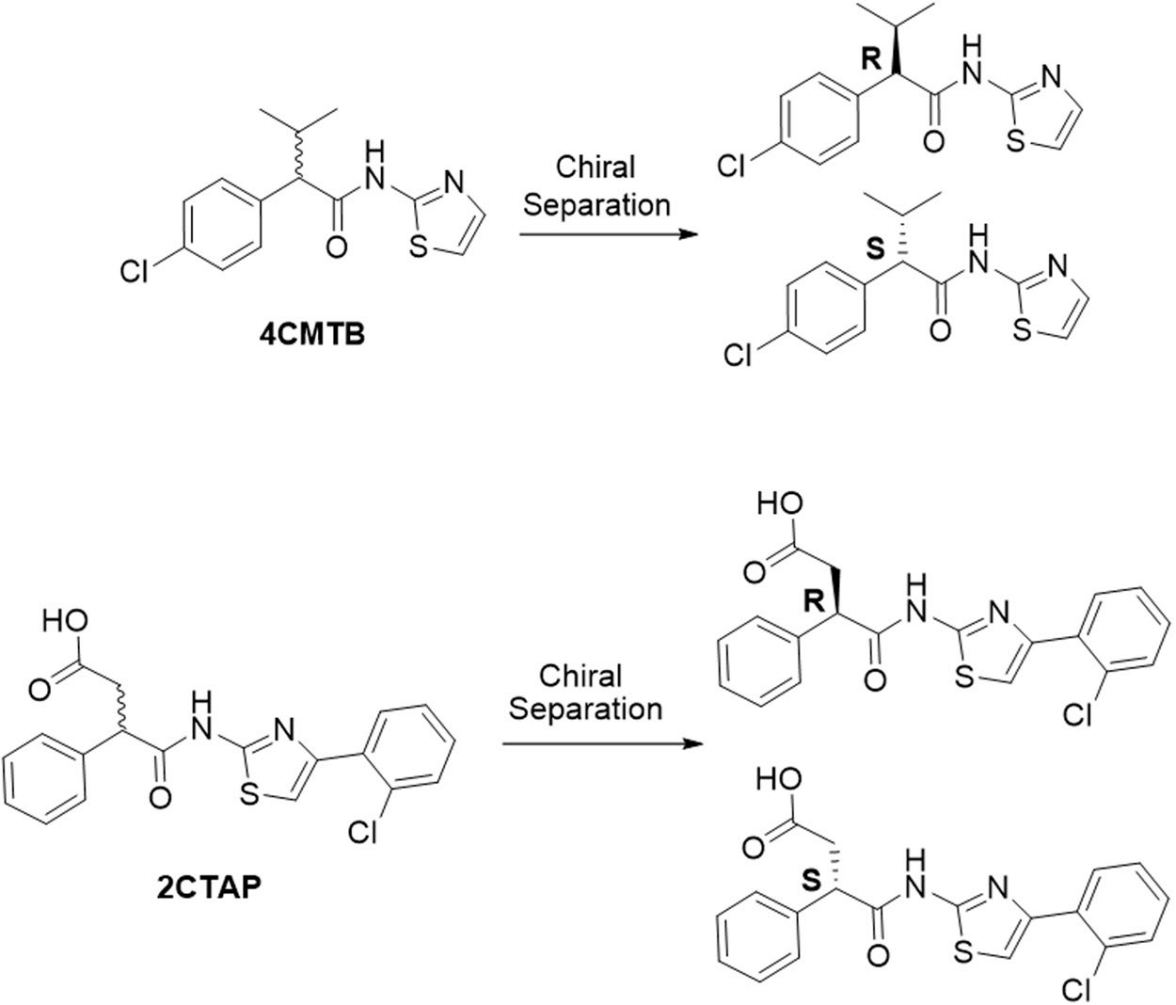
Chemical structures of 2-(4-chlorophenyl)-3-methyl-*N*-(thiazole-2-yl)butanamide (4-CMTB) and 4-((4-(2-chlorophenyl)thiazole-2-yl)amino)-4-oxo-3-phenylbutanoic acid (2CTAP) with resolved enantiomers for each.

**Figure 2:**
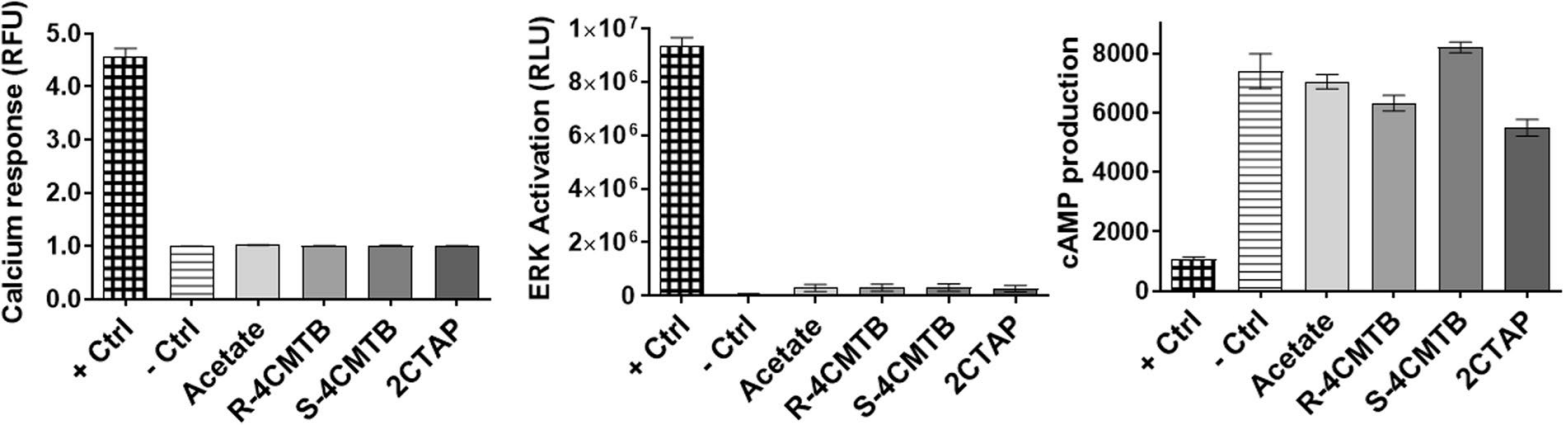
Responses for the highest concentrations of acetate, R-4CMTB, S-4CMTB and 2CTAP on parental CHO-K1 cells from left to right Ca2+ response, pERK1/2 response, Forskolin induced cAMP response. Ligand responses are plotted against positive and negative controls for each assay. Data are mean ± SEM combined from at least three independent experiments.

**Figure 3:**
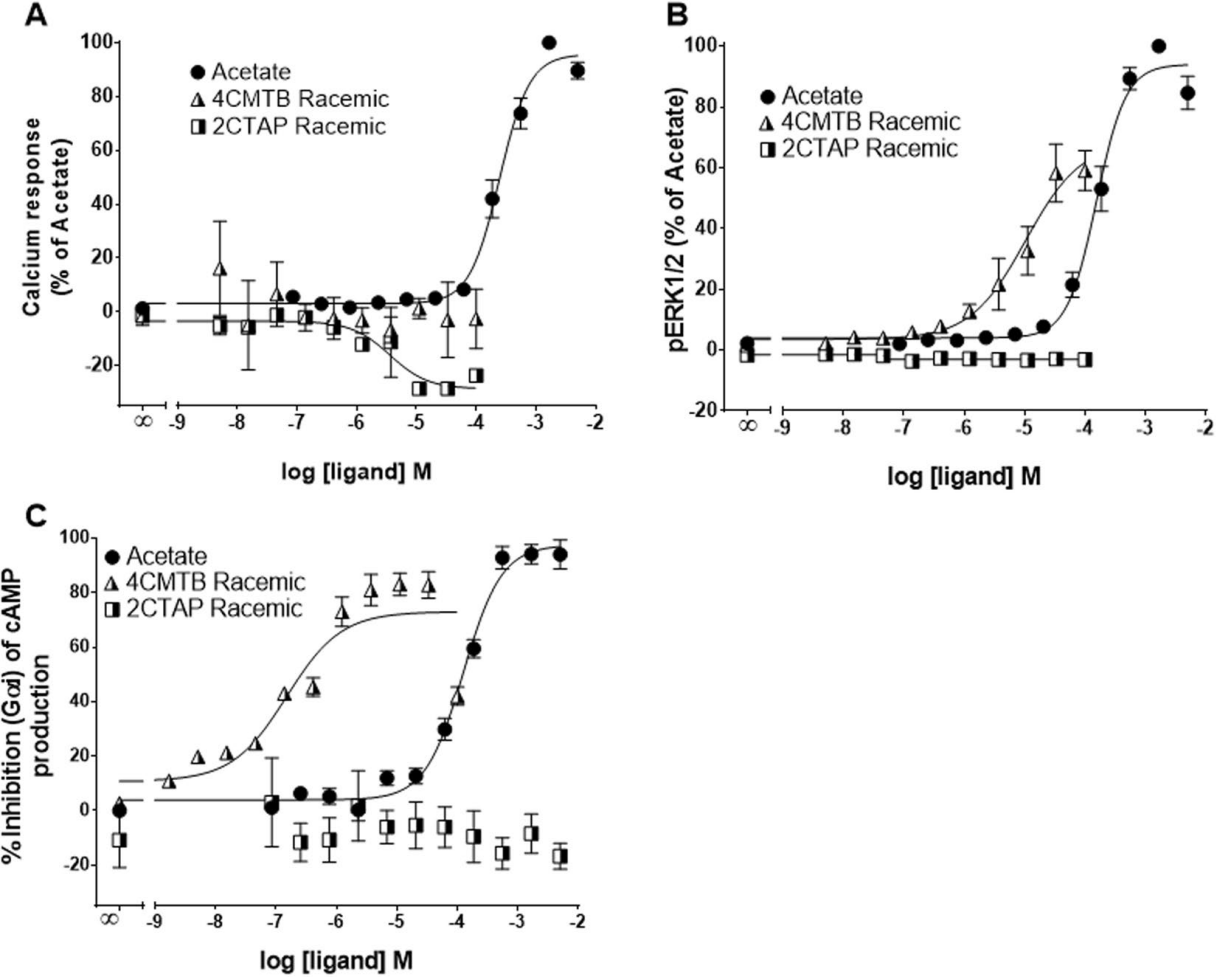
Biologic activity of 4CMTB and 2CTAP in Ca^2+^, ERK and cAMP functional assays in CHO cells stably expressing hFFA2. (**A**) Ca^2+^ release was detected using the Fluo-4-direct Ca^2+^ assay. (**B**) pERK1/2 response was measured using AlphaScreen SureFire pERK1/2 (Thr202/Tyr204) assay kit. (**C**) Inhibition of forskolin-induced cAMP measured using the LANCE Ultra cAMP assay. Data were combined from at least three independent experiments and expressed as a percentage of the maximal response from acetate.

FFA2 signals via Gα_q_, Gα_i_^2,6,15^ and β-arrestin^16^. To characterise the functional selectivity of 4CMTB and 2CTAP for FFA2, Ca^2+^, pERK and cAMP assays were carried out, with comparison to the known FFA2 agonist, acetate, in a CHO cell line stably transfected with hFFA2. The chemical structures of racemic 4CMTB (*R/S*-4CMTB) and 2CTAP (*R/S*-2CTAP) and chiral separations are shown in Fig. 1. To ensure that the responses seen in these assays were specific for FFA2 and not activating native receptors expressed on the parental CHO-K1 cell line the FFA2 ligands used in this study were continuously tested on the parental CHO-K1 cell line alongside CHO-FFA2 cells. No response was induced by acetate, 4CMTB, 2CTAP or their enantiomers in any of the assays. Responses for the highest concentration of the ligands seen in the parental CHO-K1 cells across Ca^2+^. pERK1/2 and cAMP are shown in Fig. 2. Acetate increased Ca^2+^, pERK1/2 and inhibited forskolin-induced cAMP, in a concentration-dependent manner (Fig. 3, Table 1). *R/S-*4CMTB did not induce a Ca^2+^ response, however it caused a concentration-dependent increase in pERK1/2 and inhibition of forskolin-induced cAMP, with its effects more potent but less efficacious than acetate (Fig. 3). *R/S*-2CTAP caused a concentration-dependent decrease in Ca^2+^, however, had no effect on pERK1/2 or forskolin-induced cAMP (Fig. 3, Table 2).

**Table 1:** pEC_50_ and Emax values for 4CMTB through Ca^2+^, ERK and cAMP assays.

Ligand	Assay					
	Ca^2+^		pERK1/2		cAMP	
	pEC_50_	Emax	pEC_50_	Emax	pEC_50_	Emax
Acetate	3.8	100	3.6	100	3.9	100
*R/S*-4CMTB	—	—	5.8	85	7.0	83
*R*-4CMTB	—	—	5.0	60	5.2	74
*S*-4CMTB	5.7	55	6.1	102	6.8	99
*R/S*-4CMTB + PTX	—	—	4.7	20		
*R*-4CMTB + PTX	—	—	4.7	16		
*S*-4CMTB + PTX	—	—	4.8	22		
*R/S*-4CMTB + Acetate	3.2					
*R*-4CMTB + Acetate	3.1					
*S*-4CMTB + Acetate	3.6

**Table 2:** pEC_50_ and Emax values for 2CTAP through Ca^2+^, ERK and cAMP assays.

	Ligand	Assay					
		Ca^2+^		pEC50		cAMP	
		pEC_50_	Emax	pEC_50_	Emax	pEC_50_	Emax
Agonist	Acetate	3.8	100	3.6	100	3.9	100
Agonist	*R/S*-2CTAP	—	—	—	—	—	—
	*R*-2CTAP	—	—	—	—	—	—
	*S*-2CTAP	—	—	—	—	—	—
Antagonist	*R/S*-2CTAP	6.0		4.8		—	
	*R*-2CTAP	6.6		5.6		—	
	*S*-2CTAP	5.7		4.4		—	
	*R/S*-2CTAP + Acetate	6.2					
	*R*-2CTAP + Acetate	6.6					
	*S*-2CTAP + Acetate	5.8					

### Characterisation of enantiomers of 4CMTB and 2CTAP in CHO cells stably transfected with FFA2

**Figure 4:**
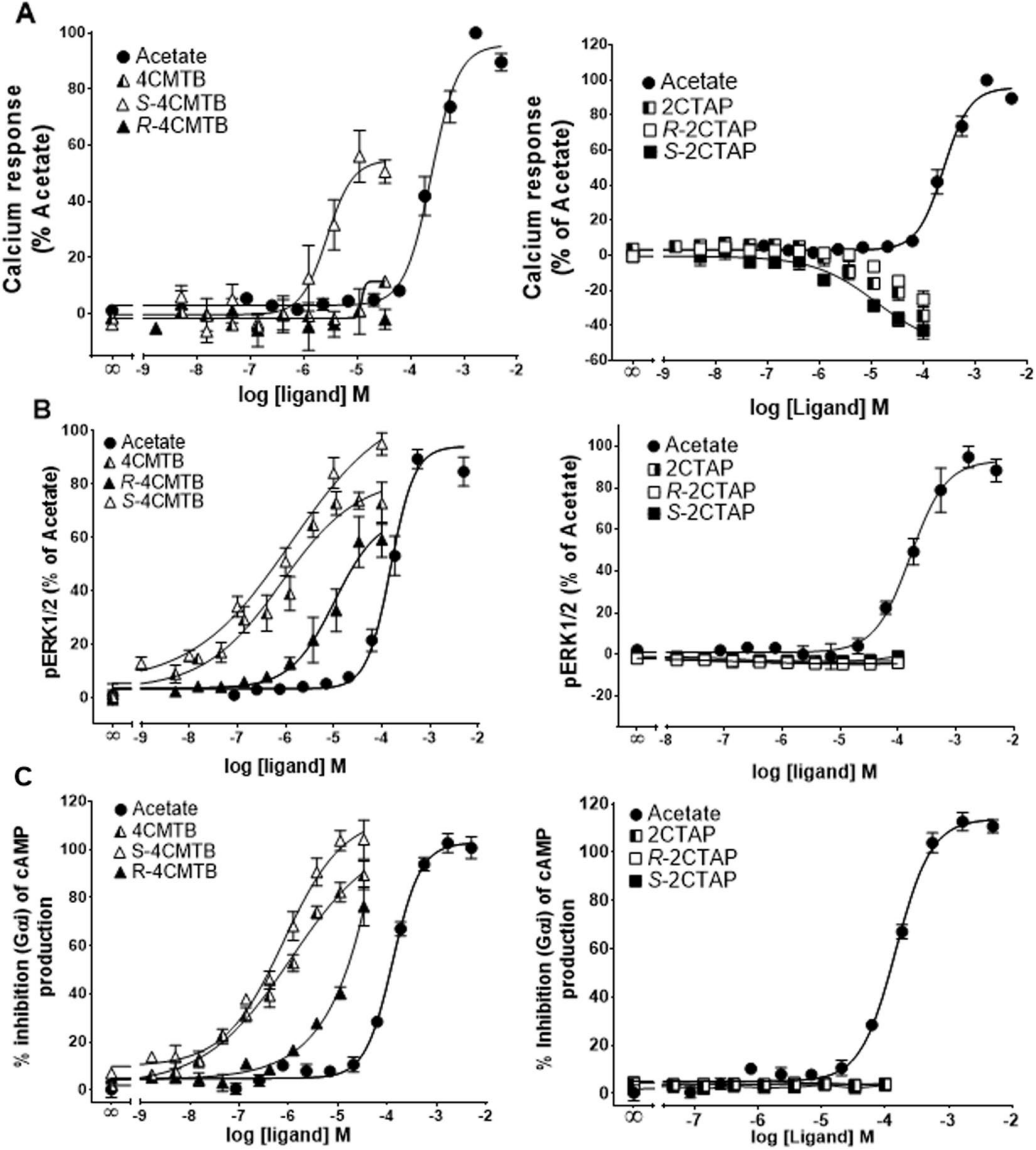
Dose response curves of 4CMTB and 2CTAP enantiomers in CHO cells stably expressing FFA2. (**A**) Ca^2+^ responses. (**B**) pERK1/2 responses. (**C**) Forskolin-induced cAMP. Data were combined from at least three independent experiments and expressed as a percentage of the maximal response from acetate.

To evaluate the functional selectivity of 4CMTB enantiomers the racemic batch was separated on a Phenomenex Lux Cellulose-2 chiral column. *S*-4CMTB elicited concentration-dependent increases in Ca^2+^, which was more potent but less efficacious than acetate, whereas *R*-4CMTB had no effect on Ca^2+^ (Fig. 4A). *S*-4CMTB and *R/S*-4CMTB had similar potency and efficacy in increasing pERK1/2 (Fig. 4B) and inhibiting forskolin-induced cAMP (Fig. 4C). *R*-4CMTB was less potent than *S*-4CMTB and *R/S*-4CMTB but more potent than acetate. *R*-2CTAP and *S*-2CTAP both decreased Ca^2+^ in a similar concentration dependent manner to *R/S*-2CTAP (Fig. 4A), however they had no effects on pERK1/2 or cAMP responses (Fig. 4B,C).

**Figure 5:**
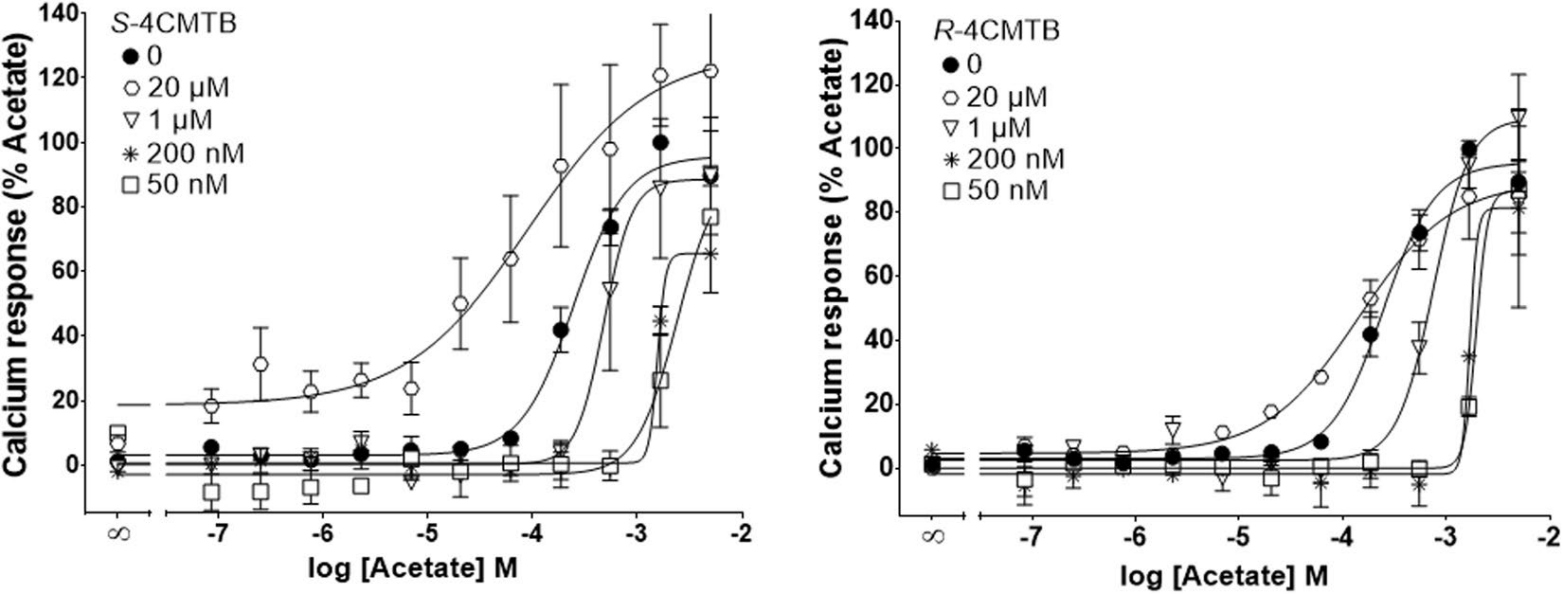
Allosteric activity of 4CMTB enantiomers in a Ca^2+^ assay. Ca^2+^ responses were measured in the presence of different concentrations of acetate with varying concentrations of *R*-4CMTB or *S*-4CMTB. Data were combined from at least three independent experiments and expressed as a percentage of the maximal response from acetate.

To study the functional selectivity of allosteric effects in a physiological setting, different concentrations of 4CMTB were tested on an acetate dose response curve. In the Fluo-4 direct Ca^2+^ assay both *S* and *R-4CMTB* were found to be negative allosteric modulators below 1 µM. At 20 µM *R*-4CMTB had a mild effect on the potency of acetate but did not increase the efficacy. At 20 µM *S*-4CMTB increased both the potency and efficacy of acetate (Fig. 5, Table 1).

### Characterisation of the functional activity of 2CTAP

**Figure 6:**
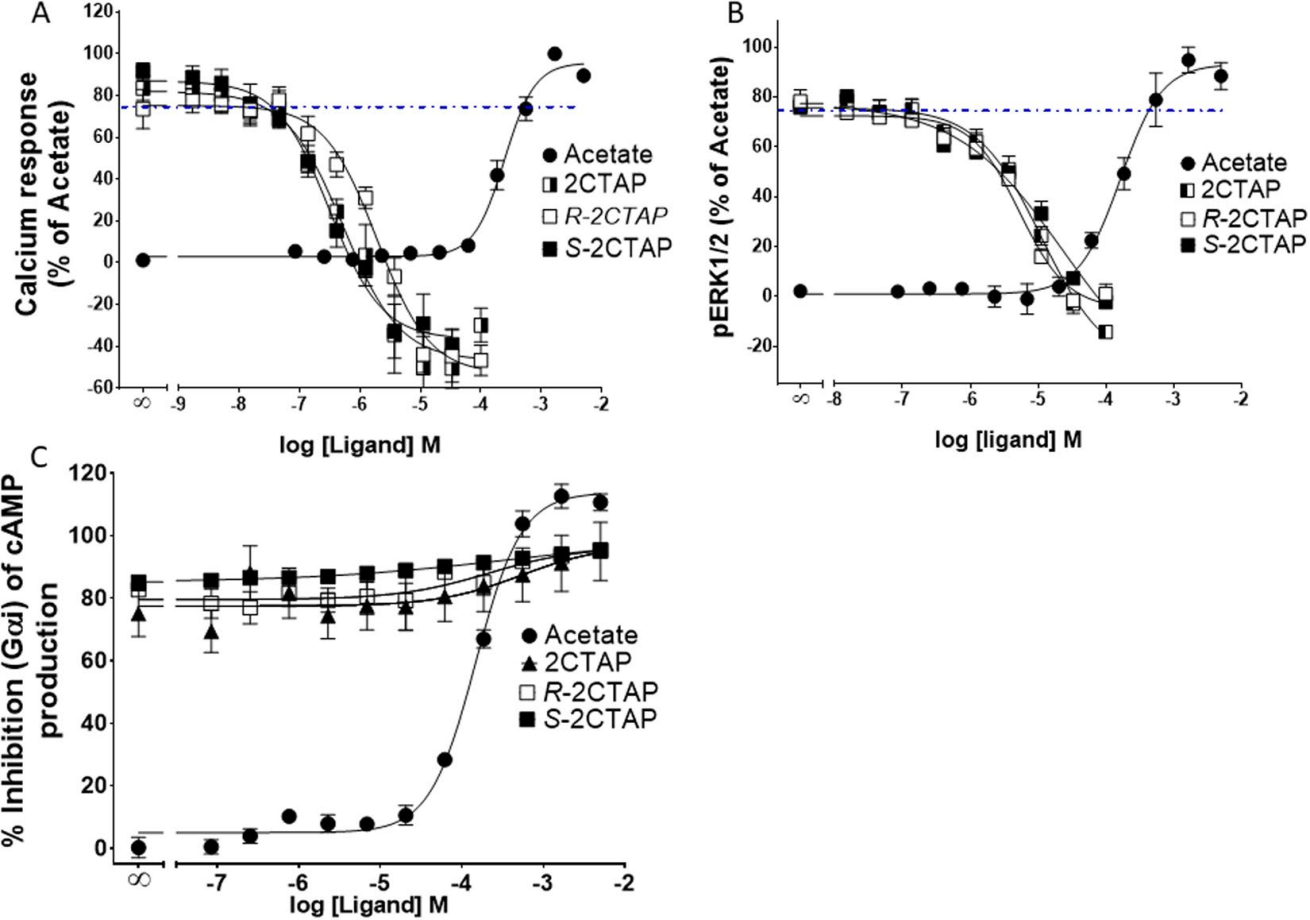
Allosteric activity of 2CTAP and the enantiomers. (**A**) Ca^2+^ responses detected in a dose dependent manner in the presence of 2 mM acetate (dotted line) show that all enantiomers of 2CTAP reduce the signal below that of the basal levels. (**B**) ERK activation by 2 mM acetate was inhibited by all enantiomers of 2CTAP. (**C**) Forskolin-induced cAMP was not inhibited by 2CTAP nor either enantiomer. Data were combined from at least three independent experiments and expressed as a percentage of the maximal response from acetate.

The functional nature of 2CTAP has not been explored beyond results from a GTPyS assay^17^. To confirm those findings and further investigate the functional nature of 2CTAP the Fluo-4 direct calcium assay, LANCE Ultra cAMP assay and a pERK1/2 assay, with a CHO cell line stably transfected with hFFA2 were utilised. *R/*S-2CTAP failed to induce intracellular Ca^2+^, phosphorylate ERK1/2 or inhibit forskolin-induced cAMP independently (Fig. 3). However, *R/S-*2CTAP appeared to inhibit constitutive Ca^2+^ levels. This effect was also seen with the *R* and *S* enantiomers with the *S* enantiomer having slightly higher efficacy over the *R* enantiomer and the recemate (Fig. 4A). Thus, indicating that 2CTAP could be an inverse agonist through the Gα_q_ pathway. To fully establish the function of 2CTAP as an allosteric modulator we tested the effects of 2CTAP dose response curves on Ca^2+^ release in the presence of 2 mM acetate (Fig. 6). 2CTAP inhibited acetate induced Ca^2+^ release in a dose dependent manner and lowered the response below constitutive levels further supporting 2CTAP as an inverse agonist through Gα_q_ (Fig. 6A). Acetate induced phosphorylation of ERK1/2 was also inhibited by 2CTAP in a dose dependent manner (Fig. 6B) suggesting that 2CTAP is an antagonist.

### The effects of functionally selective ligands in a mouse model of ischemia reperfusion injury (IRI)

**Figure 7:**
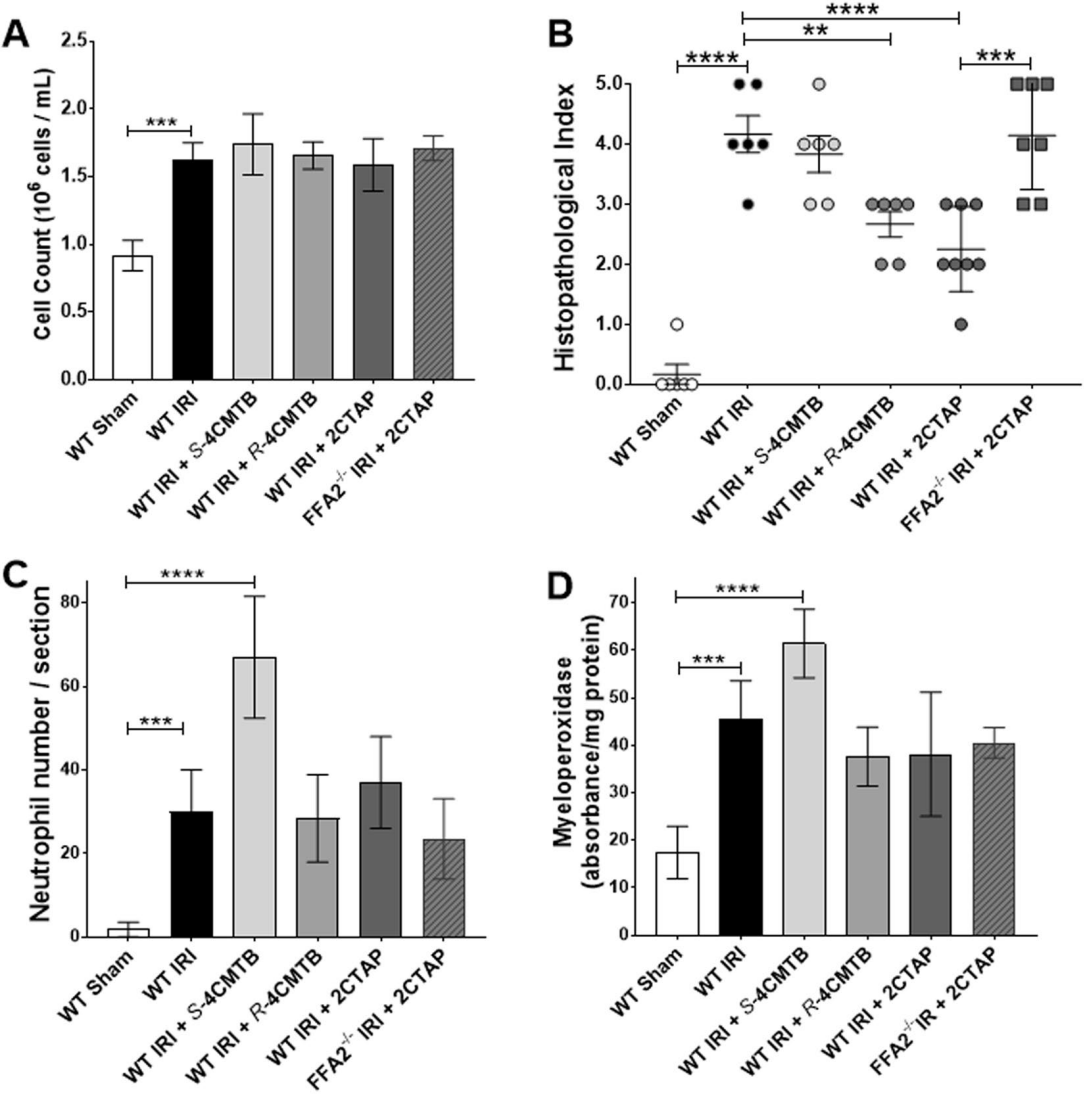
The effects of *R* and *S*-4CMTB and 2CTAP in a mouse model of intestinal IRI. (**A**) Serum neutrophil numbers. (**B**) Quantitative histopathological scores of H&E-stained intestinal sections. (**C**) Neutrophil infiltration detected by counting esterase stained neutrophils in ileum sections. (**D**) Neutrophil infiltration into the ileum measured by myeloperoxidase. Data are mean ± SEM. ****p < 0.0001; ***p < 0.001 **p < 0.01. n = 7 ± 1.

The signalling data indicates that *S*-4CMTB has a similar profile to acetate but is specific to FFA2^18^ where as 2CTAP appears to be an antagonist and thus could offer different therapeutic advantages in disease. To further advance the understanding of these ligands in disease we tested these ligands in the neutrophil dependent mouse model of superior mesenteric artery occlusion (SMOA) that represents intestinal IRI^19^. We confirmed an increase in circulating neutrophils in IRI compared to sham (Fig. 7A). Ligands dosed to sham-operated mice did not increase circulating neutrophils (Circulating neutrophil counts for sham-operated groups dosed with *R-*4CMTB = 0.81 × 10^6^ ± 0.11; *S-*4CMTB = 0.92 × 10^6^ ± 0.07; 2CTAP = 0.83 × ^1^0^6^ ± 0.09). Circulating neutrophil numbers in IRI groups dosed with *R*-4CMTB, *S*-4CMTB and 2CTAP were comparable to vehicle control IRI-groups Fig. 7A. Histological examination of haematoxylin and eosin (H&E) stained ileum sections showed normal mucosal villi in vehicle control and dosed sham-operated mice. In sharp contrast, mice subjected to IRI displayed numerous swollen haemorrhagic villi and extensive development of sub-epithelial spaces as indicated by a high histopathological score. *S-*4CMTB did not reduce villi damage however, mucosal damage was significantly reduced in WT IRI groups dosed with 2CTAP, and, although to a lesser degree, *R*-4CMTB (Fig. 7B). Neutrophil infiltration upon reperfusion is considered to significantly contribute to the damage caused during IRI^20^. Infiltration of neutrophils into the ileum was therefore examined by counting esterase-stained neutrophils and measuring myeloperoxidase (Fig. 7C and D). *S*-4CMTB increased neutrophil infiltration into the villi (Fig. 7C,D) in line with its previously shown chemotactic properties^6,13^. *R*-4CMTB and 2CTAP did not reduce neutrophil infiltration below the IRI group despite their protective effects, suggesting they may rather be modulating neutrophil function at the site of injury. The protective effects of 2CTAP were abolished in the absence of FFA2 indicating that the *in vivo* activity observed with 2CTAP is specific to FFA2 (Fig. 7).

## Discussion

Selective, allosteric, potent ligands that bind to FFA2 may have potential as therapies for inflammatory diseases. We characterised two reported FFA2 ligands (4CMTB and 2CTAP) and their enantiomers to assess their pharmacological properties at hFFA2. We demonstrated that *S*-4CMTB is a positive allosteric modulator whereas *R*-4CMTB is a functionally selective ligand that does not activate Gα_q_. 2CTAP is a functionally selective inverse agonist that inhibits constitutive Ca^2+^ levels and antagonises acetate-induced ERK phosphorylation. This study substantially extends the previous knowledge of the activity of 4CMTB and 2CTAP by characterising the enantiomers of 4CMTB and 2CTAP, relative to acetate, in CHO cells stably expressing hFFA2 and in a mouse model of intestinal IRI.

The SCFAs acetate, propionate and butyrate activate FFA2 and FFA3 and have important roles in health and disease. Notably this includes their anti-inflammatory properties that are mediated through the modulation of chemotaxis, reactive oxygen species (ROS) and cytokine production^11,14,21^. Several allosteric ligands for FFA2 have been reported that could be developed into novel therapies^17,18,22^. However, these have not been characterised across G protein pathways and assumed function is often based on a single signalling pathway or through binding studies alone. FFA2 is known to signal through Gα_i_, Gα_q_ and β-arrestin pathways^2,15,23^. Functional selectivity at orthosteric sites is a well characterised concept that has changed fundamental views on GPCR signalling^24,25^. Although functional selectivity across allosteric sites is not as well documented, it is hypothesised that allosteric ligands are more likely to have biased signalling properties, which could differ again in the presence of the orthosteric ligand^26^.

This study describes the functional selectivity of two synthetic molecules for FFA2. The phenylacetamide compound 4CMTB from Amgen is a putative ago-allosteric agonist at FFA2^18^, and the carboxylic acid derivatised variant, 2CTAP from Euroscreen, is a putative allosteric agonist at FFA2. 4CMTB has been studied across different signalling pathways with a focus on binding assays and Gα_i_ and Gα_q_ pathways^7,18^. It has been shown to be a positive allosteric modulator of FFA2^27,28^. 4CMTB is a direct FFA2 agonist in cAMP and [S35]GTPγS assays with 100- to 1,000-fold higher affinity than acetate and propionate, but with a positive modulatory effect on acetate and propionate *in vivo*^21^. Other studies have reported various other effects of 4CMTB including chemotaxis of human^6^ and mouse^13^ neutrophils, reduced proliferation of leukaemia cells^29^, mediation of intracellular signalling in adipocytes via Gα_i_^30^ and inhibition of lipolysis in differentiated 3T3L1 cells^18^. The *in vivo* observations of 4CMTB suggest that it is an ago-allosteric modulator, but mutational studies have failed to identify the binding sites of 4CMTB^18,31^.

2CTAP was patented as an allosteric agonist of FFA2 based on a binding assay^17^ with a maximal response at 60 nM in the GTPγS assay. It also increased glucose uptake in 3T3-L1 cells and inhibited lipolysis in isolated adipocytes in a concentration-dependent manner^18^. Acetate has been used as an endogenous ligand throughout these studies as it has been shown to be the most specific SCFA agonist of FFA2^6^ with no histone deacetylase activity observed^13^.

4CMTB did not induce calcium signalling in a Fluo-4 direct Ca^2+^ assay but did show positive cooperativity with acetate providing greater potency and increased efficacy over acetate alone. Surprisingly, 2CTAP did not have any agonist or ago-allosteric properties in a Ca^2+^ assay however it inhibited acetate-induced calcium responses in a dose dependent manner, suggesting that 2CTAP is an antagonist of FFA2.

The Gα_q_ pathway and intracellular Ca^2+^ levels have been reported to promote neutrophil migration^32^ and the production of ROS^33^. Neutrophils are a common cause of damage in acute inflammatory diseases and thus blocking Gα_q_ could prevent excessive neutrophil migration^32^ and reduce ROS production. FFA2 antagonists have already been reported to be able to reduce neutrophil migration^17,22,34^. The current study demonstrates that *R-*, *S-* and *R/S-*2CTAP can inhibit acetate-induced increases in Ca^2+^. In the mouse model of intestinal IRI neutrophil infiltration was not prevented by 2CTAP. However, mucosal damage was diminished in the IRI group dosed with 2CTAP and this was negated in FFA2^−/−^ mice. The mechanism for this protection could be through 2CTAP blocking FFA2 induced ROS production although further investigation is required to fully elucidate the specific mechanisms of 2CTAP on neutrophil function.

Interestingly, the allosteric activity of *R* and *S*-4CMTB was different through the Gα_q_ pathway. It is well established, unfortunately due to the thalidomide tragedy, that compounds exist as a racemic mixture of (*R*) and (*S*) enantiomers which can have different biological activity. The subtle differences in the atomic structure mean that in a calcium assay *S*-4CMTB is an ago-allosteric ligand where *R*-4CMTB does not have any intrinsic ability and appears to be a negative allosteric modulator. This is supported by the *in vivo* data which showed *R*-4CMTB did not increase neutrophil infiltration and had a protective effect in IRI in contrast to *S*-4CMTB. Unlike *R*-4CMTB and the antagonist 2CTAP, *S*-4CMTB increased neutrophil infiltration corroborating the importance of the Gα_q_ pathway in FFA2 induced chemotaxis.

pERK1/2 and cAMP are key downstream signalling pathways of FFA2. In neutrophils, activation of pERK1/2 can cause the formation of neutrophil extracellular traps^35^, and cAMP activation leads to neutrophil chemotaxis^36^. Extracellular traps have been associated with increased damage in heart and hepatic ischemia reperfusion injury^37,38^. *R*, *S*- and *R/S*-4CMTB have similar potency and efficacy for pERK1/2 and partially inhibit forskolin-induced cAMP suggesting they are Gα_i_ ago allosteric modulators. These responses are more potent than the selective FFA2 agonist acetate. 2CTAP inhibited pERK1/2 but not cAMP. Inhibition of pERK1/2 could contribute to the protective effects seen *in vivo*. Further investigation is required to fully understand the exact biological activity that these ligands have at each pathway and how this affects neutrophil function. However, we have identified 2CTAP as an antagonist that is specific at FFA2 and *R* and *S*-4CMTB which are functionally selective ligands for FFA2 and therefore present valuable tools for elucidating Ca^2+^, pERK1/2 and cAMP signalling downstream of FFA2 and the effects of FFA2 on neutrophil function.

There have been many challenges surrounding FFA2 biology, including poor potency and selectivity of ligands, altered function due to the allosteric mode of action and the fact that FFA2^−/−^ mice also have reduced expression of FFA3. *R-*, *S-* and *R/S-*4CMTB provide useful ligands to elucidate signalling bias at FFA2 at specific concentrations and *R-*, *S-* and *R/S-*2CTAP provide useful antagonists to elucidate FFA2-induced Ca^2+^ signalling in disease settings. Further studies assessing the efficacy of these compounds in rodents is warranted as some of the incomplete understanding of FFA2 biology is due to variability in ligand selectivity between human and rodent FFA2^21,39^. However, initial studies in our mouse model of intestinal IRI support the *in vitro* data carried out on hFFA2 suggesting mouse and human homologues may have similar biological function and thus further investigation of these ligands in other disease models could be beneficial.

There is a need to assess enantiomeric forms of molecules, particularly targeting hFFA2 as activation of distinct signalling pathways may provide different biological effects. 4CMTB, reinforced the often-overlooked potential of enantiomers to impart different signalling properties and potential therapeutics may go unnoticed in a racemic mixture. *S*- and *R/S*-4CMTB are agonists at FFA2 showing similar potency and efficacy for pERK1/2 and cAMP signalling, with *S-*4CMTB more potent than acetate at increasing Ca^2+^. 4CMTB and 2CTAP therefore provide useful compounds to further elucidate the translational applicability of FFA2 agonists and antagonists into mouse models of disease, and to clarify the discrepancies surrounding FFA2 biology. Unravelling the mechanisms of these pharmacologically selective ligands provides invaluable tools to further FFA2 biology and pharmacology, particularly in neutrophil-driven disease.

## Methods

### CHO-K1 cells

Chinese Hamster Ovary (CHO-K1) cells were purchased from Cellank Australia as a negative control, as they do not endogenously express FFA2. These cells were cultured in Ham’s F12, 10% FBS and 1% penicillin streptomycin. Cells were passaged when 70–80% confluent using 0.05% trypsin: EDTA in phosphate-buffered saline (PBS), pH 7.4. All cells in culture, once seeded, were incubated at 37 °C with 5% CO_2_.

### CHO-FFA2 cells

CHO-K1, stably expressing human FFA2 (CHO-FFA2) was purchased from DiscoverX Corporation. CHO-FFA2 cells were cultured in a buffer of Ham’s F12, 10% FBS, 1% penicillin streptomycin and 800 μg/mL of G418 (Geneticin) (Invitrogen Life Technologies). Cells were passaged when 70–80% confluent using 0.05% trypsin with 1 mM EDTA in phosphate-buffered saline (PBS), pH 7.4. All cells in culture, once seeded, were incubated at 37 °C with 5% CO_2_.

### FFA2 ligands

Acetate was purchased from Chem-Supply (Gillman, Australia), propionate and butyrate were purchased from Sigma-Aldrich (Missouri, USA). 4CMTB patented by Amgen^18^ and 2CTAP patented by Euroscreen^16^ were synthesized as below from purchased components.

### Synthesis of 4CMTB

**Figure 8:**
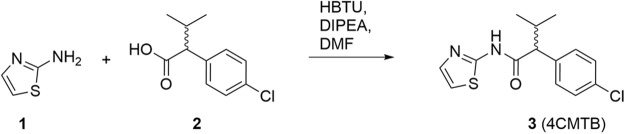
Synthetic route to 4CMTB.

4CMTB was synthesised according to the route shown in Fig. 8. where the commercially available 2-aminothiazole and 2-(4-chlorophenyl)-3-methylbutanoic acid were reacted using standard HBTU coupling with Hünig’s base in DMF. The desired amide 4CMTB was obtained in 70% yield after recrystallization and data are consistent with literature precedent.

### Synthesis of 2CTAP

**Figure 9:**
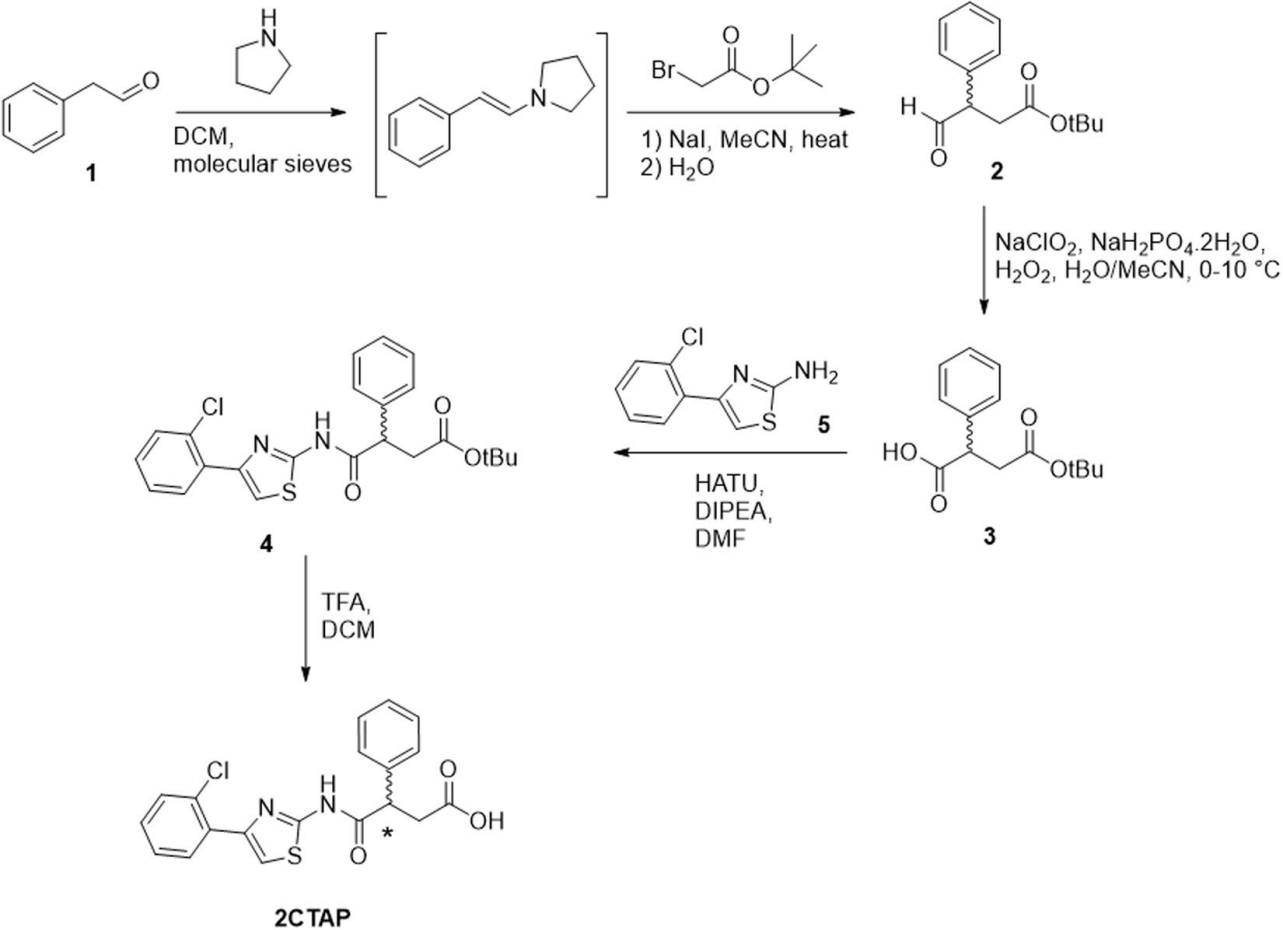
Synthetic route to 2CTAP.

2CTAP was synthesised according to the route shown in Fig. 9. Phenylacetaldehyde (1) was converted to the enamine via reaction with pyrrolidine under anhydrous conditions following the procedure of Belanger *et al*.^40^. Subsequent reaction with t-butyl bromoacetate and sodium hydride followed by hydrolysis of the enamine gave the desired aldehyde (2), tert-butyl 4-oxo-3-phenylbutanoate in moderate yield over the two steps. Potassium permanganate was initially used as an oxidant to convert the aldehyde to the corresponding acid (3) however yields were exceptionally low, and reactions did not proceed cleanly. The milder Pinnick oxidation procedure^41^ gave conversion to 4-(tert-butoxy)-4-oxo-2-phenylbutanoic acid (3) in 59% yield. 4-(2-chlorophenyl)thiazol-2-amine (5) was synthesized using standard Hantsch thiazole synthesis. Coupling of carboxylic acid (3) with the 2-aminothiazole (5) using standard HBTU coupling gave the protected product (4) with 93% yield after purification using column chromatography. Subsequent deprotection using trifluoroacetic acid gave 2CTAP in quantitative yield with spectra in agreement with literature. The racemic 2CTAP was separated into the two enantiomers using chiral column chromatography. Chiral LC-MS analyses were conducted using an Agilent Technologies 1200 Series Instrument with a G1316A UV-Vis detector (λ = 210 and 254 nm), 1200 Series ELSD and 6110 quadrupole ESI-MS, using Phenomenex Lux Cellulose-2 column (4. 6 × 250 mm, 250 Å~4.6 mm, 3 μm, flow rate 1 mL/min, room temperature). The eluents were 0.05% formic acid in water (A) and 0.05% formic acid in ACN (B). Using a 30 min isocratic method set at 60% mobile phase B. The Chiral HPLC purifications were achieved using a Gilson PLC 2020 Personal Purification System using a Phenomenex Lux Cellulose-2 column (21.2 × 150 mm, 150 Å~21.2 mm, 5 μm, flow rate 20 mL/min, room temperature). The eluents were 0.1% formic acid in water (A) and 0.1% formic acid in ACN (B). Using a 30 min isocratic method set at 60% mobile phase B. Samples were dissolved in chloroform and optical rotation was determined on an automatic polarimeter. The enantiomers were formulated as the sodium salt to aid their aqueous solubility in subsequent testing.

### Ca^2+^ flux assay

Fluo-4 Direct assay was preformed according to manufacturer’s instructions with additional wash steps and read on a Fluorescent Imaging Plate Reader (FLIPR) (Molecular Devices, California). Briefly, 10,000 cells were seeded/well in 10 μL of media in a 384 well block with a clear bottom plate and incubated overnight at 37 °C. An equivalent volume of 2x Flou-4 Direct calcium reagent was added to each well and incubated for 30 min at 37 °C and a further 30 min at room temperature. Ligand addition was carried out on the FLIPR instrument whilst fluorescence was measured at excitation 494 nm and emission at 516 nm for five minutes to enable capture of the fast calcium response.

### ERK1/2 phosphorylation assay

To measure pERK1/2 we utilised the high throughput AlphaScreen SureFire pERK1/2 (Thr202/Tyr204) Assay Kit (PerkinElmer)^42^. Detection of pERK1/2 is enabled by immuno-sandwich capture of endogenous pERK1/2 in cell lysates. Antibody-coated AlphaScreen beads generate a highly amplified signal when near due to binding of pERK1/2. Cells were seeded at 50,000 cells/well in a 96 well tissue culture plate and incubated for 24 hours at 37 °C. Cells were then incubated in serum free media overnight. Test compounds were added to the cells and incubated for 10 min at room temperature before cells were lysed and assayed for pERK1/2 using the AlphaScreen-based detection kit according to the manufacturer’s protocol (PerkinElmer).

### cAMP assay

The LANCE Ultra cAMP homogeneous time resolved resonance energy transfer (TR-FRET) immunoassay (PerkinElmer) provides a highly sensitive and robust method for the detection of cAMP. Briefly, cells were re-suspended in stimulation buffer (1 x HBSS, 5 mM HEPES, 0.5 mM IBMX, 0.1% BSA (pH7.4)) and transferred to 384-proxiplate (PerkinElmer) at a density of 2000 cells/well. Cells were stimulated with agonist and forskolin (5 μM), where appropriate, and incubated for 30 min. EucAMP tracer and ULight-anti-cAMP solutions were added to the cells and incubated for 1 hour at room temperature. Time- resolved FRET signals were measured after excitation at 320 nm on the EnVision. All data were normalized to the functional response obtained with 1.67 mM sodium acetate.

### Conventional and FFA2 knockout mice

Specific pathogen-free (SPF) conventional C57BL/6J (WT) mice were obtained from the Australian Research Council (ARC) and maintained at The University of Queensland Biological Resources Animal Facilities under specific pathogen-free conditions.

FFA2^−/−^ knockout mice were obtained from Monash University and bred at The University of Queensland Biological Resources Animal Facilities under SPF conditions. Male mice 10–12 weeks weighing 20–30 g were used for all experiments. All experiments were performed in accordance with the guidelines and approval of The University of Queensland Animal Ethics Committee.

### Intestinal Ischemia Reperfusion Injury Model

Anesthetized mice underwent laparotomy followed by superior mesenteric artery occlusion by a loop of ligature to induce nontraumatic intestinal ischemia. After 35 minutes the ligature was removed to allow for a reperfusion period of 150 minutes. Sham-operated mice underwent the same surgical procedure except for the superior mesenteric artery occlusion. Mice were orally gavaged with ligand 48 hours, 24 hours and 1 hour prior to surgery. Mice were euthanised and small intestinal tissue or whole blood [collected in 1 mg/mL EDTA and 0.1 mg/mL nafamostat mesylate (FUT-175)] was collected for further analysis.

### Data and statistical analysis

All data is expressed as mean ± SEM from at least three independent experiments. Data analysis and curve fitting were carried out using the GraphPad Prism v6.0 software package (GraphPad Software, California). Concentration-response data were fit to four parameter sigmoidal concentration-response curves. All statistical analysis of curve fit parameters was carried out by independently fitting the data from triplicate experiments and comparing the resulting curve fit values by t-test or one-way-ANOVA as appropriate. Statistical differences between curves was analysed using an F-test. All statistical analysis of *in vivo* work was carried out using one-way anova with Tukey’s multiple comparison.

## Data Availability

The datasets generated during and/or analysed during the current study are available from the corresponding author on reasonable request.
